# Gaming in China Before the COVID-19 Pandemic and After the Lifting of Lockdowns: a Nationwide Online Retrospective Survey

**DOI:** 10.1007/s11469-022-00792-3

**Published:** 2022-03-31

**Authors:** Qiuxia Wu, Tao Luo, Jinsong Tang, Yunfei Wang, Zhenzhen Wu, Yueheng Liu, Wei Chen, Qijian Deng, Yanhui Liao

**Affiliations:** 1 Department of Psychiatry, and National Clinical Research Center for Mental Disorders, The Second Xiangya Hospital of Central South University, Changsha, 410011 Hunan China; 2 The Treatment Center for Addiction, Jiangxi Mental Hospital, Nanchang, 330029 Jiangxi China; 3 Department of Psychiatry, Sir Run Run Shaw Hospital, School of Medicine, Zhejiang University, Hangzhou, 310016 Zhejiang China; 4 Ningbo Kangning Hospital, Ningbo, 315201 Zhejiang China; 5 Key Laboratory of Medical Neurobiology of Zhejiang Province, Hangzhou, 310016 China

**Keywords:** Gaming behavior, COVID-19, Time spent on gaming, Distress, Internet gaming disorder

## Abstract

With the lockdown and social distancing during the outbreak of coronavirus disease 2019 (COVID-19), gaming has become a popular leisure activity. This study aimed to explore changes in gaming behavior after the lifting of COVID-19 lockdowns and risk factors for increased gaming behavior. This online retrospective study included 5268 gamers. A total of 5% gamers scored 32 or higher on the 9-item Internet Gaming Disorder Scale—Short-Form (IGDS9-SF), suggesting diagnosis of internet gaming disorder (IGD). Over one-third of gamers reported an increase in time spent on gaming per day after the lockdowns were lifted. Logistic regression analysis revealed that gamers who were female, students, experienced stress, or scored higher on IGDS9-SF were more likely to spend more time on gaming per day after the lifting of lockdowns. These findings highlighted the needs for more effective coping strategies or interventions to prevent excessive gaming, especially for females and students.

The ubiquity of video games has been impacting people’s lives in many ways. Although gaming could be beneficial from several aspects, including cognition, emotion, and brain structure and function (Denilson et al., 2019; Granic et al., 2014), excessive gaming could increase the risk of internet gaming disorder (IGD), which might affect gamers’ physical and mental well-being, and even lead to social dysfunction (Saunders et al., 2017). Since the beginning of the coronavirus disease 2019 (COVID-19) pandemic, various mitigation measures, such as lockdowns and social distancing, have been taken, which increased the level of people’s psychological distress to some extent (Qiu et al., 2020). With social restrictions, video gaming has become a coping strategy against distress, as people could connect with each other without face-to-face meeting (Giardina et al., 2021). The growth rate of global gross domestic product (GDP) in 2020 is − 3.4% (World Bank, 2022), leading a collapse in global economic activity. However, the revenues of game industry in 2020 increased 23% than that of 2019 (Newzoo, 2021), indicating the increase of gaming activities.

There are several factors associated with gaming behavior. Previous studies reported that males were more engaged in gaming than females (Griffiths et al., 2004; Winn & Heeter, 2009). Psychological distress (Wu et al., 2018), including stress (Canale et al., 2019; Yu et al., 2018), depression (Yen et al., 2019), and anxiety (Adams et al., 2019; Männikkö et al., 2020), has also been reported to be associated with gaming behavior and even IGD.

A brief report about changes in addictive behaviors during the pandemic showed that almost half of the participants had increased dependence on the internet, and 16.6% of them spent more time on the internet (Sun et al., 2020). A study that included 128 Indian college students reported that around 50% of the participants had increased gaming behavior during the lockdown (Balhara et al., 2020). A study including 80 Japanese participants with IGD or excessive use of the internet or gaming also found that participants spent more time on the internet and gaming during the stay-at-home period, as compared to before COVID-19 (Higuchi et al., 2020). Although the pandemic in China is generally stable, small-scale local epidemic occurred in some areas from time to time. The pattern of gaming behavior after the pandemic still remains unknown. Assessment of patterns of gaming behavior before the pandemic and after the lifting of lockdowns may provide insight into the development of more effective coping strategies to prevent the development of problematic gaming or IGD.

Little is known about the changes in gaming behavior through the COVID-19 period in China. In the present study, we aimed to explore the impact of the pandemic on gaming behavior among Chinese gamers. Studies conducted in other countries reported an increase in gaming behavior or gaming interest through the COVID-19 pandemic, which might be related to isolation or emotional distress (Giardina et al., 2021; Paschke et al., 2021; Sharma et al., 2021; Xu et al., 2021). We hypothesized that gamers might spend more time on gaming after the lifting of lockdowns, as compared to that before the pandemic. Previous studies also reported an association between gaming and distress (i.e., stress, anxiety, and depression) (Adams et al., 2019; Männikkö et al., 2020; Yen et al., 2019). It is widely reported COVID-19 related distress during the pandemic (Ahmed et al., 2020; Shi et al., 2020), which would sustain after the lifting of lockdowns. Two recent studies also reported distress persists after the lifting of lockdowns (Alharbi et al., 2022; Haucke et al., 2021). We also hypothesized that the increase in gaming behavior might be associated with distress.

## Methods

### Participants and Recruitment

This study was conducted from May 7, 2020 to August 3, 2020 in China, when lockdowns had been lifted for most cities in China (Wikipedia, n.d.). Wuhan, as the first city to lock down, was the last one to lift its lockdown on April 8, 2020. Only the capital of Xinjiang Province started lockdown during the period of our survey. People could go shopping, dine in the restaurants, and return to their work with green health code and strict prevention measures (see the supplementary materials). The survey was advertised on websites and social media, such as WeChat, and was performed via Questionnaire Star, a professional online survey service. Participants aged 18 or older were eligible for this nationwide online cross-sectional survey, while those unable to read texts in simplified Chinese or unwilling to provide electronic consent form were excluded. The survey was anonymous. Participants were asked whether they had any gaming experience during the past 12 months. Gaming was defined as activities of playing video games with electronic devices, including but not limited to computers, laptops, and other devices (such as mobile phones, tablets, PS4, and Switch). All the questions were required to be answered before final submission to ensure that there is no missing data in this survey. This study is a part of a large online study on people’s mental health and changes in gaming behavior, alcohol consumption (Wang et al., 2020a, b), and social media use (Luo et al., 2021) in China after the lifting of lockdowns.

### Measures

#### Social Demographics

Participants’ basic data, including gender, age, level of education, occupation, marital status, and residence (rural, urban), were collected.

#### Gaming Variables

The time spent on gaming was measured using a Timeline Followback questionnaire, which included (1) average time spent on gaming (in minutes) per day before the pandemic and after the lifting of lockdowns and (2) average number of days engaged in gaming per week before the pandemic and after the lifting of lockdowns. Information related to gaming behavior, such as the age of starting playing games and duration of gaming, was assessed. Gaming disorder was assessed using the Chinese version of the 9-item Internet Gaming Disorder Scale—Short-Form (IGDS9-SF) (Pontes & Griffiths, 2015). The IGDS9-SF is a widely used tool based on the diagnostic criteria for IGD in the Diagnostic and Statistical Manual of Mental Disorders-5 (DSM-5) (American Psychiatric Association, 2013). In IGDS9-SF, each item is rated on a 5-point Likert scale, with a score ranged from 1 (never) to 5 (very often). The optimal cutoff for a positive diagnosis of IGD is 32 in the context of China (Qin et al., 2020).

#### Distress

Symptoms of stress, anxiety, and depression were assessed using the Chinese version of the 21-item Depression Anxiety Stress Scales (DASS-21) (Lovibond & Lovibond, 1995), which showed good reliability and validity (Chan et al., 2012). It is a self-report scale that consists of three subscales measuring depression, anxiety, and stress. Each subscale consists of 7 items, all of which are rated on a 4-point Likert scale with a score from 0 (never) to 3 (almost always). The total score of each subscale needs to be multiplied by two. The ranges of scores for normal, mild, moderate, severe, and extremely severe stress/anxiety/depression were set as follows: 0–14, 15–18, 19–25, 26–33, and 34 + for stress; 0–7, 8–9, 10–14, 15–19, and 20 + for anxiety; and 0–9, 10–13, 14–20, 21–27, and 28 + for depression.

### Quality Control

The response of survey could be submitted only once for each WeChat ID. The IP address, WeChat ID, and responses to each item of the questionnaire were checked to make sure each participant submitted his/her response only once. This study found no duplicate submission.

### Statistical Analysis

Statistical analyses were performed using R 3.6.1 in Rstudio 1.2.5001 (R Core Team, 2019). Demographic data were presented as median (interquartile range) or frequency (percentage). The characteristics and gaming behavior between gamers with increased time spent on gaming per day after the lifting of lockdowns (the increase group) and gamers without increase in their gaming time per day (the non-increase group) were compared. As the variables were not normally distributed, we performed nonparametric statistical tests, including chi-square test and Wilcoxon rank sum test. Differences in gaming behavior between the time before the pandemic and after the lifting of lockdowns were analyzed using paired samples Wilcoxon test, and potential risk factors (i.e., demographic factors, psychological factors) contributing to increased gaming time per day were identified using logistic regression analysis. The level of statistical significance was set at *p* < 0.05 (two-tailed).

## Results

### Demographic Characteristics

A total of 5268 participants completed the survey. The median age of these participants was 27 years; over half of them were male (52.6%), unmarried (58.1%), and employed (62.8%). Most of them had an education level of high school and above (93.4%) and lived in the urban area (94.5%). Demographic characteristics of the overall sample and the two groups are presented in Table 1. A total of 5% gamers scored 32 or higher on the IGDS9-SF, suggesting for IGD. Gamers in the increase group were more likely to be younger, female (49.6%), students (47.1%), unmarried (67.6%), having an education level of college or above (95.0%), living in the rural area (6.8%), younger when they initiated game playing, as well as having higher rates of stress (21.6%), anxiety (38.5%), and depression (34.6%) symptoms and higher scores of IGDS9-SF.

**Table 1:** Demographic characteristics

	Overall sample (*n* = 5268)	The non-increase group (*n* = 3318, 63.0%)	The increase group (*n* = 1950, 37.0%)	*p* value ^a^
Age	27 (22, 35)	29 (23, 27)	25 (21, 32)	< 0.001
Gender				0.012
Male	2773 (52.6)	1791 (54.0)	982 (50.4)	
Female	2495 (47.4)	1527 (46.0)	968 (49.6)	
Education				< 0.001
High school or lower	350 (6.6)	253 (7.6)	97 (5.0)	
College or higher	4918 (93.4)	3065 (92.4)	1853 (95.0)	
Occupation				< 0.001
Students	1785 (33.9)	867 (26.1)	918 (47.1)	
Unemployed	173 (3.3)	114 (3.4)	59 (3.0)	
Employed	3310 (62.8)	2337 (70.4)	973 (49.9)	
Marriage				< 0.001
Married	2206 (41.9)	1574 (47.4)	632 (32.4)	
Unmarried	3062 (58.1)	1744 (52.6)	1318 (67.6)	
Residence				0.002
Urban	4978 (94.5)	3161 (95.3)	1817 (93.2)	
Rural	290 (5.5)	157 (4.7)	133 (6.8)	
Mental health problem^b^ (%)				
Stress	1136 (21.6)	660 (19.9)	476 (24.4)	< 0.001
Anxiety	2026 (38.5)	1242 (37.4)	784 (40.2)	0.049
Depression	1821 (34.6)	1102 (33.2)	719 (36.9)	0.008
Age of starting playing games	15 (11,18)	15 (11,19)	15 (10,18)	0.001
Duration of gaming (year)	9 (3,16)	10 (3,17)	9 (4,15)	0.749
IGDS9-SF total score	17 (11,23)	16 (10,21)	18 (14,25)	< 0.001
IGDS9-SF score ≥ 32	264 (5.0)	146 (4.4)	118 (6.1)	0.01

Data are presented as *n* (%) or median (interquartile range)

*DASS* 21-item Depression Anxiety Stress Scale, *IGDS9-SF* 9-item Internet Gaming Disorder Scale—Short-Form

^a^Significantly different, *p* < 0.05 (Wilcoxon two samples rank test for continuous variables and *χ*^2^ test for categorical variables)

^b^Mental health problems were rated as mild, moderate, severe, and extremely severe

### Gaming Behavior Before COVID-19 and After the Lifting of Lockdowns

Gaming behavior before COVID-19 and after the lifting of lockdowns for the overall sample and the two groups is presented in Table 2. Among all the respondents, 37.0% reported an increase in gaming time per day. For the overall sample, the time spent on gaming per day increased by 30–40 min (*p* < 0.001), and the number of days engaged in gaming per week increased by 3–4 days (*p* < 0.001) after the lifting of lockdowns. For the increase group, the time spent on gaming per day after the lifting of lockdowns increased by 30–90 min (*p* < 0.001), and the number of days engaged in gaming per week increased by 3–5 days (*p* < 0.001). For the non-increase group, the time spent on gaming per day and the number of days engaged in gaming per week decreased after the lifting of lockdowns (*p* < 0.001).

**Table 2:** Gaming behavior before the pandemic and after the lifting of lockdowns

Variable	Before COVID-19	After the lifting of lockdowns	*p* value
Time spent on gaming per day (minutes)			
Overall	30 (10, 60)	40 (10, 100)	< 0.001
Non-increase group	30 (1, 60)	20 (0, 60)	< 0.001
Increase group	30 (15, 60)	90 (50, 160)	< 0.001
Days engaged in gaming per week (days)			
Overall	3 (1, 5)	4 (1, 7)	< 0.001
Non-increase group	3 (0, 6)	2 (0, 5)	< 0.001
Increase group	3 (2, 5)	5 (3, 7)	< 0.001

*COVID-19* coronavirus disease 2019

### Inter-Group Differences in Gaming Behavior Before COVID-19 and After the Lifting of Lockdowns

Differences in gaming behavior between the increase group and the non-increase group before COVID-19 and after the lifting of lockdowns are shown in Table 3. Both groups spent approximately 30 min per day and 3 days per week playing games before the pandemic, but the interquartile ranges of the two groups were different. It was shown that the increase group spent more time on gaming per day and engaged in gaming for more days per week after the lifting of lockdowns, as compared to the non-increase group.

**Table 3:** Differences in time spent on gaming per day and days engaged in gaming per week before COVID-19 and after the lifting of lockdowns between the two groups

	Non-increase group (*n* = 3318, 63.0%)	Increase group (*n* = 1950, 37.0%)	*p* value
Time spent on gaming per day (minute)			
Before COVID-19	30 (1, 60)	30 (15, 60)	< 0.001
After the lifting of lockdowns	20 (0, 60)	90 (50, 160)	< 0.001
Days engaged in gaming per week			
Before COVID-19	3 (0, 6)	3 (2, 5)	< 0.001
After the lifting of lockdowns	2 (0, 5)	5 (3, 7)	< 0.001

*COVID-19* coronavirus disease 2019

### Risk Factors of Increased Gaming Behavior

As shown in Table 1, there were significant differences in age, gender, education level, occupation, marital status, and residence status between the increase group and the non-increase group. The logistic regression model showed that female (*p* = 0.029, OR = 1.18, CI [1.02, 1.38]), students (*p* = 0.007, OR = 1.76, CI [1.17, 2.66]), stress (*p* = 0.045, OR = 1.24, CI [1.01, 1.54]), and higher total score of IGDS9-SF (*p* < 0.001, OR = 1.04, CI [1.02, 1.05]) were significantly associated with increased time spent on gaming (Table 4).

**Table 4:** Risk factors of increased gaming behavior by logistic regression analysis

Variable	OR	CI	*p* value
Gender (female)	1.18	(1.02, 1.38)	0.029
Occupation (employed)	0.67	(0.45, 1.01)	0.056
Occupation (students)	1.76	(1.17, 2.66)	0.007
Education (college or higher)	1.34	(0.98, 1.84)	0.063
DASS			
Stress ^a^	1.24	(1.01, 1.54)	0.045
Anxiety ^a^	0.85	(0.71, 1.03)	0.097
IGDS9-SF total score	1.04	(1.02, 1.05)	< 0.001

*OR* odds ratio, *CI* 95% confidence interval, *DASS* 21-item Depression Anxiety Stress Scale, *IGDS9-SF* 9-item Internet Gaming Disorder Scale—Short-Form

^a^Mental health problems were rated from moderate to extremely severe

## Discussion

To the best of our knowledge, this was the first study to assess the changes in gaming behavior through the COVID-19 period in China. The present study found that, compared with the time before the pandemic, the postlockdown era witnessed more gaming behavior in China. This study found 5% gamers might meet the diagnostic criteria for IGD. For gamers who spent more time on gaming per day after the lifting of lockdowns, they were also engaged in gaming for more days per week, as compared to those who did not spend more time playing games per day. Gamers who were female, students, suffering from stress, or with high scores of IGDS9-SF were more likely to spend more time on gaming after the lifting of lockdowns.

Compared with the time of before COVID-19, both the time spent on gaming per day and the number of days engaged in gaming per week increased after the lifting of lockdowns in the overall gamer population. In the present study, 37.0% of the respondents reported an increase in time spent on gaming per day, and 44.7% of the potential gamers with IGD reported an increase in time spent on gaming per day. A similar survey among 128 Indian college students reported that 59% of the participants had increased gaming behavior (Balhara et al., 2020). And a study that included 80 treatment seekers in Japan reported that 52.5% had an increase in time spent on online gaming (Higuchi et al., 2020). The higher rates of increased gaming behavior in these studies might be attributed to different sample sizes and different populations studies; in the study in India, 14.84% of participants might have IGD, according to DSM-5 and IGDS9-SF, while 70% of participants in the study in Japan were diagnosed with gaming disorder according to the International Classification of Diseases 11th Revision (ICD-11), with 20% of them engaged in excessive gaming. In our study, which included 5268 participants, only 5.0% of them might be diagnosed with IGD according to IGDS9-SF.

The increase in gaming behavior after the lifting of lockdowns might be attributed to several factors. First, people were not able to engage in face-to-face social interactions as well as other outdoor activities. Therefore, online gaming became a major leisure activity during this special period, as it could help people socialize without breaking the stay-at-home order; this has been confirmed by a report that, during the pandemic, the engagement in multiplayer gamed increased more prominent than single-player games (Vuorre et al., 2021). Second, the fear of both COVID-19 per se and lockdowns could increase people’s distress, such as stress, anxiety, and depression, which could directly affect people’s gaming behavior and indirectly lead to escape from reality (Ballabio et al., 2017; Wang et al., 2020a, b). It was also reported that gaming could be helpful in emotion regulation (Király et al., 2015; Villani et al., 2018), especially when outdoor activities were limited. Third, as many people had to pause their work or had to work remotely from home, they might have much more spare time to play games.

This study found that the prevalence of stress, anxiety, and depression symptoms among Chinese gamers were 21.6%, 38.5%, and 34.6%, respectively. According to some previous studies in China, the prevalence of anxiety ranged from 22.0 to 36.4% (Ahmed et al., 2020; Li et al., 2014; Ni et al., 2020; Ran et al., 2020; Wang et al., 2020a, b), the prevalence of depression ranged from 19.2 to 47.1% (Ahmed et al., 2020; Li et al., 2014; Ni et al., 2020; Ran et al., 2020; Wang et al., 2020a, b), and the prevalence of stress was 32.1% (Wang et al., 2020a, b). The reasons for these discrepancies might be different sample sizes, scales used for assessments, or different time of evaluation. The fear of COVID-19, long-term lockdowns, uncertainty caused by COVID-19, financial issues, and the uncertain future might also lead to high prevalence of stress, anxiety, and depression.

The median time spent on gaming per day and the median number of days engaged in gaming per week before COVID-19 were similar between the two groups, but the interquartile ranges for the two variables were slightly higher in the increase group. The increase group spent more time on gaming per day (more than four times) and was engaged in gaming for more days per week after the lifting of lockdowns, as compared to the non-increase group, while the non-increase group spent slightly less time on gaming per day and were engaged in gaming for fewer days per week; these changes were significant. The present study revealed that gamers who were female, students, suffering from stress, or having a higher IGDS9-SF total score were more likely to spend more time on gaming per day after the lifting of lockdowns.

Even though the proportions of female and male gamers in the increase group were almost the same, female gamers were more likely to spend more time on gaming after the lifting of lockdowns. Previously, it was believed that females tended to spend less time playing video games than males (Ratan et al., 2015); however, some studies showed opposite results (Kuo et al., 2012). One reason that female gamers tended to spend more time on gaming than their male counterparts might be their stronger needs for social interactions during this period. According to some previous studies, females were more engaged in social interactions, while males were more achievement-oriented (Kuo et al., 2012; Veltri et al., 2014; Williams et al., 2009). Another reason for more time spent on games by females might be the stress they felt during COVID-19, as females are reported more likely to be involved in avoidance behaviors to cope with stress (Hassouneh & Brengman, 2014). Furthermore, females are required to do more obligatory activities, especially housework, which leaves them less free time to play games before the COVID-19 pandemic. During COVID-19, both males and females had to stay home, resulting in females having relatively more free time to play games.

Students were more likely to spend more time on gaming after the lifting of lockdowns. Students with more free time are more likely to spend more time playing games (Winn & Heeter, 2009). During the lockdowns, students had more flexibility in their study and were unable to have a part-time job, which might lead to more spare time spent on gaming. In addition, the results of online learning might not be satisfactory as compared to that of face-to-face classes (Faidley, 2018), which might increase the fear of academic year loss. A previous study reported that the fear of academic year loss was correlated with distress during COVID-19 (Hasan & Bao, 2020), which could also lead to an increase in gaming time.

Gamers with increased gaming behavior showed higher degrees of stress, anxiety, and depression than those without increased gaming behavior. In this study, we also found a positive correlation between stress and increased time spent on gaming per day. Previous studies also found a positive correlation between perceived stress and gaming time (Canale et al., 2019). Moreover, previous study also reported that playing video games could help reduce stress (Roy & Ferguson, 2016), which might, to some extent, explain the positive relationship between stress and increased time spent on gaming in our study. However, as the motivation of gaming was not investigated in this study, whether the primary motivation of gamers’ spending more time on gaming was to cope with stress remained unknown.

This study also found that the increase in time spent on gaming was positively correlated with the total score of IGDS9-SF. Gamers with a higher total score of IGDS9-SF were more likely to be diagnosed with IGD; according to one of the criteria for IGD in DSM-5, i.e., tolerance, these gamers had the need to spend increasing amount of time playing games (American Psychiatric Association, 2013). Previous studies also reported a positive correlation between time engaged in gaming and the diagnosis of IGD (Rho et al., 2016; Wang et al., 2014), which is consistent with our findings.

There are several limitations in our study. First, we conducted this study online through convenience sampling, which might lead to biases, reducing the representativeness of the population. Second, as the participant information of gaming behavior was self-reported, their time spent on gaming might be underestimated. Third, this is a cross-sectional retrospective survey, which required participants to recall their time spent on gaming before COVID-19; therefore, the result might be less accurate due to memory biases. Finally, the types of games or motivations of gaming were not investigated in this study, which precluded us from identifying the types of games (such as e-sports games, action games, etc.) that attracted more attention or identifying their main motivation of gaming; therefore, it was difficult for us to determine whether the gaming behavior was in fact harmful or beneficial. Despite the limitations, the present study was strengthened by a large sample size, and the results still implicate the needs for more effective coping strategies against stress through the COVID-19 period.

## Conclusion

In this study, we have found that the prevalence of IGD was 5% after the lifting of lockdowns. Compared with before the pandemic, there was an overall increase in gaming behavior after the lifting of lockdowns in China. The increase in time spent on gaming was positively correlated with female, students, stress, and the total score of IGDS9-SF. These findings highlighted the need for attention to the development of effective coping strategies or interventions to prevent excessive gaming, especially for females and students.

## Data Availability

The dataset used and/or analyzed during the current study is available from the corresponding author on reasonable request.
